# Usefulness of Dismissing and Changing the Coach in Professional Soccer

**DOI:** 10.1371/journal.pone.0017664

**Published:** 2011-03-22

**Authors:** Andreas Heuer, Christian Müller, Oliver Rubner, Norbert Hagemann, Bernd Strauss

**Affiliations:** 1 Institute of Physical Chemistry, University of Muenster, Muenster, Germany; 2 Institute of Organic Chemistry, University of Muenster, Muenster, Germany; 3 Institute of Sports Sciences, University of Kassel, Kassel, Germany; 4 Institute of Sports Sciences, University of Muenster, Muenster, Germany; University of Pittsburgh, United States of America

## Abstract

Whether a coach dismissal during the mid-season has an impact on the subsequent team performance has long been a subject of controversial scientific discussion. Here we find a clear-cut answer to this question by using a recently developed statistical framework for the team fitness and by analyzing the first two moments of the effect of a coach dismissal. We can show with an unprecedented small statistical error for the German soccer league that dismissing the coach within the season has basically no effect on the subsequent performance of a team. Changing the coach between two seasons has no effect either. Furthermore, an upper bound for the actual influence of the coach on the team fitness can be estimated. Beyond the immediate relevance of this result, this study may lead the way to analogous studies for exploring the effect of managerial changes, e.g., in economic terms.

## Introduction

Fred Everiss, responsible for the soccer team of West Bromwich Albion (UK) coached his team over 46 years (1902–1948) without any interruption. This is probably the all time world record for coaches in professional soccer. In Germany, for instance, Volker Finke is the record holder. He coached the professional soccer team of SC Freiburg for almost 16 years (1991–2007) without interruptions (German record), although due to the relegation into the Second German soccer league his team had to leave the Premier German Soccer league (the so called “Erste Bundesliga”, established 1963) four times. However, such loyalty is very unusual in professional team sports. Frequently, the usual response to a continuing series of recent lost matches is to dismiss and replace the coach mid-season. For example in the German Bundesliga the club “Eintracht Frankfurt” is leading in dismissing a coach during mid season (20 times in 47 years of the German Premier soccer league). Fired coaches are often hired by competitors who also dismissed the coach. For example, Gyula Lorant as well as Joerg Berger are the most often dismissed head coaches in the German Bundesliga (six times each).

The reason to fire a coach mid-season [1] is often due to disappointed expectations in comparison to the team wage bill [2] and to the widespread assumption of clubs, fans, and the media that changing the coach has a major positive effect on a subsequent team's performance (one-way causality hypothesis) [3]. This is opposed to the Ritual Scapegoating Hypothesis, i.e. dismissing the coach will have no effect on a team's performance (the nil hypothesis) [3]. The latter follows the assumption that a coach has only a small impact on the performance of the team which the coach is responsible for.

Already in 1964 [3] preferred the hypothesis of ritual scapegoating. However, a closer inspection of their empirical findings in professional Baseball could not clearly support any of their presented hypotheses. Not surprisingly, whether mid-season coach dismissals have effects on the subsequent team performance has long been a subject of controversial discussions, mainly in the Sport Sciences [4] and Economic Sciences as well [1], [5].

Many of these studies focused on coach dismissals in professional soccer in different national leagues. These studies disagree with respect to the final result as well as the used research design. Partly these results have to be questioned due to design problems like a sub-optimal choice of the performance criterion [1], [6]–[15], the use of a very small data basis (e.g., Dutch soccer [8], [9], Spanish soccer [10]), missed control teams [1], [8], [10], or a biased choice of control teams (English soccer [11], [12], German soccer [13]–[15], Dutch soccer [16]).

## Methods

### Team Fitness in Soccer from a Statistical Perspective

- Heuer and Rubner [7] have recently shown theoretically that the mathematically optimal measure of a soccer team's fitness is the goal difference (ΔG). Therefore, to optimize the predictability it is essential to use ΔG rather than the number of points or the rank as a characteristic of the team fitness (as almost always used by the studies mentioned above, a rare exception is [8]). Stated differently, the number of points contains a larger random contribution than the goal difference. Qualitatively, the superiority of goal differences as compared to points expresses the fact that a 5∶0 and a 1∶0 win is counted identically in terms of points although in general this difference indicates the presence of different fitness values for both teams. Quantitatively, the identification of random contributions can be achieved via a straightforward correlation analysis of subsequent sets of matches (e.g., by comparing the first and the second half of the season).

Most importantly, a team's fitness remains just about constant throughout a season. Any variations during the season are due to temporal fluctuations (like weather conditions, red cards) whereas systematic variations mainly occur between different seasons [6], [7]. This observation already gives a hint to formulate our main hypothesis in line with [3] that changing the coach during the season is useless and would have no effects in the subsequent team performance. Using optimized statistical approaches to avoid the design problems mentioned above these questions will be answered in this work. Additionally, to classify these mid-season dismissal effects on subsequent performances we will also analyze the effects of changing the coach between seasons.

### Analysis of Coach Dismissals (CDs)

We analyze the Premier German soccer league (as we already mentioned, the so-called German “Erste Bundesliga”) which started in the season 1963/64. We consider all mid-seasonal coach dismissals (CDs) for all 46 seasons until 2008/09. Almost in each season every team has to play 34 games (except the three seasons 1963/64, 64/65 as well as 1991/1992). The entire data set covers 14,018 games. Since during the first decades of the Bundesliga several matches have been adjourned due to weather conditions etc. it is essential to take into account the correct order of matches for each team. The key procedure of our approach can be summarized as follows

1. To be able to quantify possible fitness variations due to the CD we require that before and after the CD the team plays at least m = 10 matches in that season, i.e. 10≤t_CD_≤24 where t_CD_ is the match day just before the CD. During the m = 10 matches before the CD no other CD is allowed. Our final data basis contains 154 CDs out of 361 mid-seasonal CDs in total. To first approximation the CDs are equally distributed in the time interval 10≤t_CD_≤24 with an average value of around 17.
2. To quantify the effect of a CD we choose an appropriate control group. For a specific CD event, occurring after match day t_CD_ (by construction t_CD_≥10), we identify all events where some other or the same team during any season displays a similar goal difference (more specifically with a difference of the goal difference ΔG between control team and CD team in the interval [0.185,−0.215]) during t_CD_ subsequent matches and has still at least 10 matches to play after this time interval. The minor asymmetry of the selection interval for control teams guarantees an identical average value of ΔG of control and CD teams and just reflects the Gaussian-type distribution of ΔG -values around zero [7]. We use always normalized goal differences (per match). In this way we obtain approximately 100 control teams per CD, except for a single extreme case in the year 1965/66 where no control teams could be found. Additionally, we have chosen a control group by two separate conditions. First, during the matches 3 to 10 before the CD event the deviation of the average goal difference ΔG between control team and CD team had to be in the interval [0.196,−0.204] and second, during the two matches before the CD event (matches 1 and 2) a per-match-deviation of the goal difference by ±0.5 was allowed. The reason for these different choices is discussed in the main part.
3. Going beyond most previous studies we have also corrected the home/away-asymmetry [7], [8], i.e. the match results are projected on the fictive results in a neutral stadium, in order to extract the respective team fitness without the home/away-bias. More specifically, we have substituted ΔG by ΔG±Δh (−: home match; +: away match) where Δh (>0) denotes the average home advantage. It turns out that the home advantage depends on the season, but is independent of the specific team [7].

Our procedure implies some important methodological aspects that have to be kept in mind:

1. The value of m = 10 has been selected by the condition that the final result displays a minimum error. In case of a larger interval the number of CDs would be smaller, in case of a smaller interval the characterization of the team fitness would be worse.
2. A few times it occurs that within the m = 10 matches a new coach is already replaced by another coach. Sometimes this is planned (in case of a caretaker coach) or is the consequence of successive bad performance. As implied by our approach we have in that case incorporated the first CD but not the second one. This is motivated by the fact that otherwise we cannot judge the team quality during the short time (less than m matches) between the first and the second CD. In any event, our setup implies that the results exactly hold for all CDs where the coach was active for at least m matches.
3. Previous studies (see above) have restricted the control group to teams which did not dismiss the coach during the relevant period. This, however, introduces a bias towards a more positive expectation because teams with a bad future performance tend to be excluded. To overcome this statistical problem it is essential to use unbiased control groups.
4. The identification of control teams via all t_CD_ matches before the CD is motivated by our previous observation that the change of the team fitness during the season is neglible so that as many matches as possible should be taken into account for the estimation of the team fitness. However, based on the subsequent results we will conclude that a minor modification of the selection process might be appropriate. In any event, this will be discussed further below.

### Analysis of Changes of Coaches (CCs)

We have also studied all cases where a coach was changed (as a regular change or a dismissal) during the summer break. This event is denoted as CC (change of coach). We have considered those 141 cases (starting 1966/67) where the corresponding team played in the German Premier League in both seasons before and after the CC. Here we start somewhat later in order to have enough seasons to estimate the team fitness before the CC (see below).

An important aspect for the CC analysis deals with the prediction of the expected outcome of a season. If during one season the goal difference is given by ΔG (old) the expected average fitness F(est) in the next season can be consistently estimated via F(est) = c_F_+d_F_ ΔG (old) [7]. Here F(est) can represent the expected goal difference or the number of points in the new season. The parameters c_F_ and d_F_ are calculated from a regression analysis for all teams which are not relegated. An even better estimator is obtained by averaging (for all teams where this is possible) the outcome over the previous three years with weighting factor 1.0, 0.7 and 0.5 for the determination of ΔG (old). These parameters have been estimated by optimizing the prediction process. If a team was not playing in the Bundesliga in the second and/or third last season, these seasons were just omitted from the calculation of ΔG (old). Note that our results are insensitive to the specific choice of these weighting factors.

## Results

### CD: Analysis of Possible Effects

**Figure 1:**
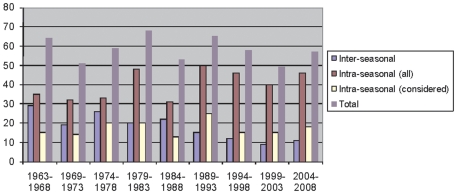
The number of CD and CC events. In particular we show the intra-seasonal CD events after the 10^th^ match and before the 25^th^ match.

The temporal evolution of CD and CC events is explicitly shown in Fig. 1. Interestingly, the total number does not show any significant time dependence. It seems, however, that the number of CC events was larger during the initial period of the Bundesliga whereas at the same time the number of CD events during the initial or final period of the season was smaller. This might be a consequence of the increased presence of media and the corresponding pressure to act in case of a bad performance.

**Figure 2:**
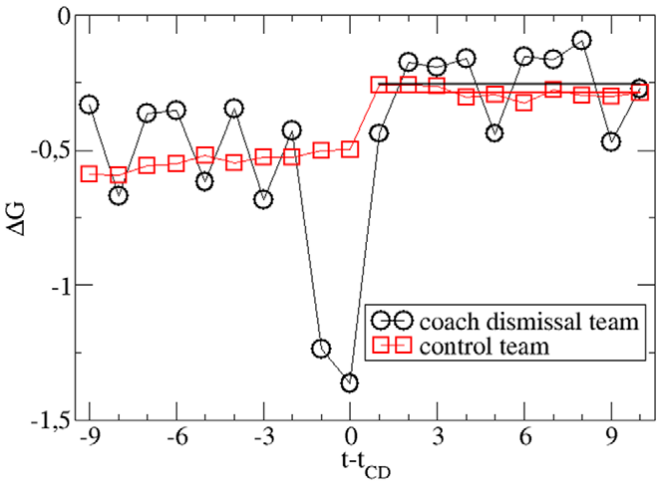
Comparison of the CD (coach dismissal) with the control teams based on the average goal difference. The time axis is shifted with respect to the time of the CD (occurring directly after match day t_CD_) to enable comparison of different events. The average values for the prediction period are included as solid lines. No effect of the CD is present within statistical errors.

In Fig. 2 we show the goal difference of an average CD team vs. time (measured in units of matches). There is a naive interpretation of this plot. First the teams, which later on will dismiss the coach, display an average value of ΔG = −0.5. Then the fitness further deteriorates down to ΔG = −1.3 which prompts the CD. Afterwards the average value of ΔG is −0.25, suggesting a significant improvement.

As already noted in literature [1], [8], [10]–[15] a group of teams with an average negative goal difference will on average also have experienced bad luck. After the selection procedure, i.e. in the prediction period (final 10 matches), any positive or negative random effects will average out. To quantify this effect we analyse the performance of the control teams as introduced in the method section. By our construction we obtain an identical average value of ΔG of control and CD teams ((ΔG = −0.539±0.002 and ΔG = −0.539±0.035, respectively. Time resolved average ΔG -values are also displayed in Fig. 2. For the prediction period we obtain an average ΔG -value of −0.257±0.044 for the CD teams and of −0.287±0.002 for the control teams, yielding Δ (ΔG) = 0.030±0.044, supporting the nil hypothesis. A more detailed error analysis which takes into account the statistical uncertainty of ΔG in the selection period, yields a slightly larger statistical error of 0.046 as compared to 0.044. With an optimistic estimation of a residual improvement of Δ(ΔG) = 0.030+0.046 = 0.076 our result amounts to a total improvement during half a season, i.e. 17 matches, of ΔG≈1.3.

Repeating this analysis for different values of m, i.e. different time intervals to define the selection and prediction period, the nil hypothesis is supported for all choices, albeit with larger statistical errors. An objective approach to judge the size of this effect is to compare the square of this maximum possible improvement with the variance of the fitness distribution which is 0.27 (see also [7] for a similar value determined for the last 23 seasons). Thus we obtain 0.076^2^/0.27 = 0.02. This again clearly shows that any possible improvement is absolutely negligible. Using a different measure of the effect size, as standard in statistical literature, yields a similarly small value [17].

This apparent improvement in Fig. 2 is known as regression towards the mean [13]–[15]. Qualitatively, this effect reflects the fact that a subgroup which is selected based on a negative accomplishment during a finite time interval will seemingly improve in the future. This is just a direct consequence of the presence of statistical fluctuations and is fully reflected by the behavior of the control teams. In the present case it can be expressed as the ratio r_ΔG_ of the average ΔG value in the prediction period and the ΔG value in the selection period. For the control teams one empirically obtains r_ΔG_ = 0.53. Previous work has developed a general formula stating the r_ΔG_ is approximately equal to 1/(1+f/t_CD_)<1 with f≈13; see [7]. With this expression at hand we can perform a consistency check of our approach. Additionally taking into account the distribution of t_CD_ values as well its average value of 17 the relevant factor here is c(13.5, 17)≈0.56 which is indeed close to 0.53. The slight variation of f reflects the difference between <1/t_CD_> and 1/<t_CD_>.

Please note that there is no gradual improvement during the m matches after the CD event. First, this result is consistent with the general observation that the team fitness does not change during the season. Second, this also implies that the cases where a carekeeper coach is replaced after less than m = 10 matches does not yield a further significant positive (or negative) shift.

We have repeated the analysis by restricting ourselves to the last 23 years of the Bundesliga. Here we find Δ (ΔG) = 0.08±0.06. Within the error bars this result is identical to that of the whole period and is thus again compatible with the nil hypothesis. Thus, there is no significant time dependence in the efficiency of CD events.

**Figure 3:**
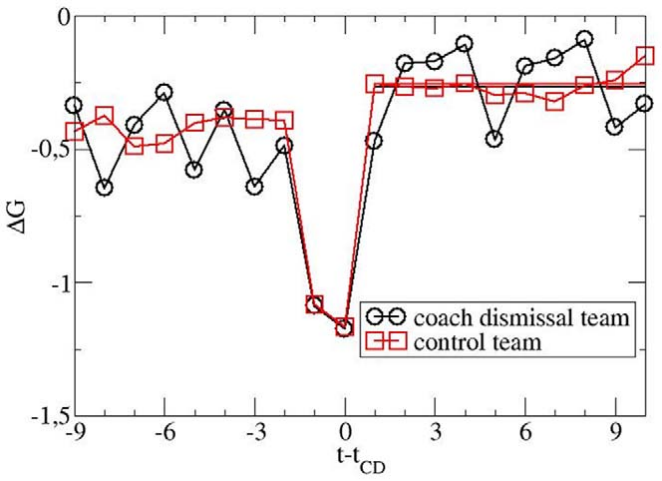
Analogous representation as in Figure 2, but with the additional constraint that the control teams also display correspondingly bad results during the two matches before the CD event. Again no effect of the CD is present.

Interestingly, the CD teams play worse during the last two matches before the CD event. Thus one might speculate that the CD event at least helps to stop this emerging negative streak. This hypothesis can be checked by selecting control teams which also have two worse results at the end of the selection period (see above for details). The results are shown in Fig. 3. Except for 14 CD teams it was always possible to find appropriate control teams, albeit with a smaller number (due to the more detailed constraints). This shows up in larger fluctuations. Again the average results in the prediction period are basically identical. This result is compatible with our previous finding [7] that two consecutively lost matches are not sufficient to identify the beginning of a negative streak (in contrast to four consecutively lost matches).

Furthermore we checked that the CD events are not related to any effects of the home/away-asymmetry. Since we have corrected out this asymmetry no effects should be present. However, we explicitly checked that within statistical noise the number of home/away and away/home matches before the CD event is nearly equal and the fraction of two subsequent home or two subsequent away matches before the CD event is both less than 7%.

**Figure 4:**
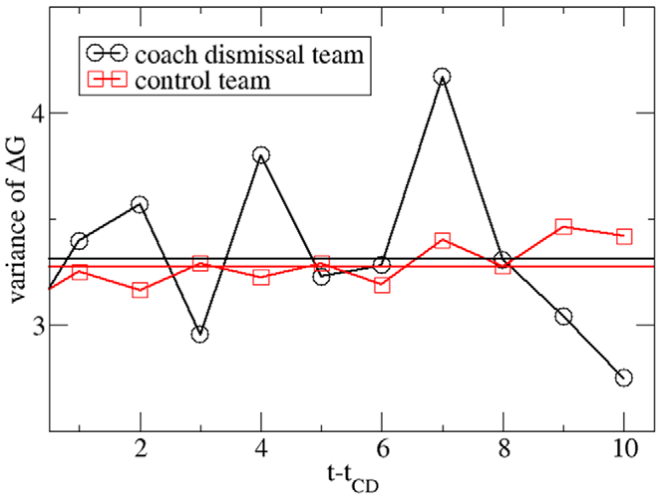
Comparison of the CD teams with the control teams based on the variance of the goal differences. In analogy to Figure 1 the average values over the prediction period are given as solid lines. Again no effect is present.

The results, reported so far, deal with the average effect of a CD event. In particular they are still compatible with the hypothesis that the CD has a positive effect for some teams and a negative effect for other teams. This can be tested by analyzing the variance of ΔG -values. Results are shown in Fig. 4. The variance increases by 0.05±0.1. This result is compatible with a zero effect. Of course, the value of 0.05 would still allow for the (extreme) scenario that half of the CD events result in an improvement of ΔG≈0.2 (≈√0.05) and the other half in a deterioration of ΔG≈–0.2. This explicitly shows that the resulting effect, if present at all, is very small effect.

In practice one is particularly interested in points P rather than in the goal difference ΔG. Because of the important implications of our results we have repeated the same analysis as in Fig. 2 by using points to characterize the fitness of teams. To standardize all games beginning in 1963 we have always used 3 points for a win and 1 for a draw following the worldwide established FIFA rules. It should be noted, that in the German Premier League 2 points were used for a win until 1994/95.

**Figure 5:**
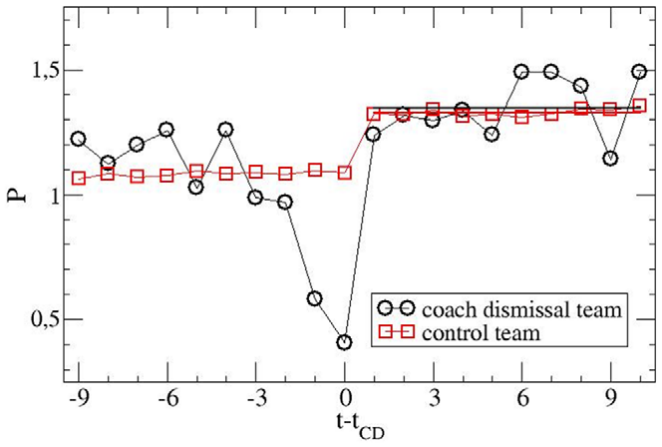
Analogous to Figure 2, using points rather than the goal difference as the observable of interest. Again no effect of the CD is present within statistical errors.

As seen in Fig. 5 the qualitative behavior is fully identical as discussed in the context of Fig. 2. The average values in the prediction period are P = 1.347±0.036 and P = 1.329±0.004 for the CD teams and the control teams, respectively. Their difference reads ΔP = 0.018±0.036. Note that ΔP = 0.018 per match corresponds too much less than one point per half season. In any event, the nil hypothesis is fully supported.

However, comparisons of the results for ΔG and P explicitly show that the information content of the goal difference is by far superior. As shown in [7] an approximate scaling of ΔG to P values can be performed by using a factor of approx.1.6 (see [7]). Indeed, this factor is approximately recovered when comparing Δ (ΔG) = 0.030 with ΔP = 0.019. However, the relative statistical error is significantly larger for ΔP, i.e. 0.2, as compared to Δ(ΔG), i.e. 0.13. Furthermore, also the strong apparent fitness decrease during the two matches before the coach dismissal is significantly more pronounced for ΔG as compared to P. This is partly due to the fact that points are (trivially) bounded from below whereas no such bound exists for ΔG.

### Motivation for a CD

A further important question deals with the motivation to dismiss a coach. Naturally, an unsatisfactory performance is expected to be the main reason. As already discussed above the data in Fig. 2 suggest that beyond this general performance argument (see below for a closer discussion) the occurrence of two bad results trigger the dismissal of the coach. This observation has consequences for the consistency of our approach. Based on our previous results [6] we expect that fitness fluctuations are very small during a season. Due to the relative shifting of the data (via t–t_CD_) we systematically identify two matches where the teams just had particular bad luck. It is consistent to exclude these two matches from the fitness estimation of a team because these two data points are biased. As a consequence the control teams on average should have the same ΔG for t–t_CD_<−1.

This argument can be rationalized with a simple example. In the “dice throwing premier league” a coach is dismissed after 2 times throwing a 1. Of course, in principle all teams have equal properties (average fitness 3.5). However, if the 10 matches before a CD event were analyzed exactly in analogy to our procedure one finds an average fitness of 3.3. The reduction is due to the systematic inclusion of the final two results with a 1. Thus, the fitness estimate is lower than the true fitness of 3.5. Excluding the last two results for the CD from the analysis yields a fitness value of 3.6. Now the value is larger as the true fitness because in our approach no second (1,1)-pair is allowed to occur during the 10 matches before the CD event. Thus we conclude that a better fitness estimate is obtained if we omit the two matches before the CD event. However, since this estimation would be slightly too optimistic, the optimum estimation lies in between both approaches (with and without the final two matches) as exemplified above.

Adapting the choice of control teams to this condition (omission of the last two matches) the average value of ΔG in the selection period reads −0.431 instead of −0.539. Correspondingly the optimized set of control teams also plays better in the prediction period (−0.235 instead of −0.287). Thus the effect of the CD gives rise to a negative value of Δ(ΔG) = −0.022±0.048 rather than Δ(ΔG) = 0.030±0.046 (as mentioned above). As a consequence our finding of a nil effect is further corroborated by this self-consistently modified procedure. As discussed in the previous paragraph for general reasons the “true” value is expected to lie between the original (0.030) and the new estimate (−0.022) which even better agrees with the nil hypothesis.

It is to be expected that beyond this triggering effect also the performance in the whole season is unsatisfactory. To quantify this effect we determine the expected number of points in a season P(est) as well as the expected goal difference ΔG(est) for all CD teams with the procedure, introduced in the method section. Then one can assess the degree of frustration of a team from comparison with the actual outcome. For this comparison we choose the number of points, i.e. P(true) – P(est), since this observable is relevant for managerial decision processes. Since the CD does not change the fitness of the team we can use the outcome of the total season to get an optimum statistical accuracy. To obtain an even more specific correlation we additionally correlate the difference P(true) – P(est) with ΔG(est), the latter representing the fitness of a team. In this way we can distinguish between the motivation of a CD for good and bad teams.

**Figure 6:**
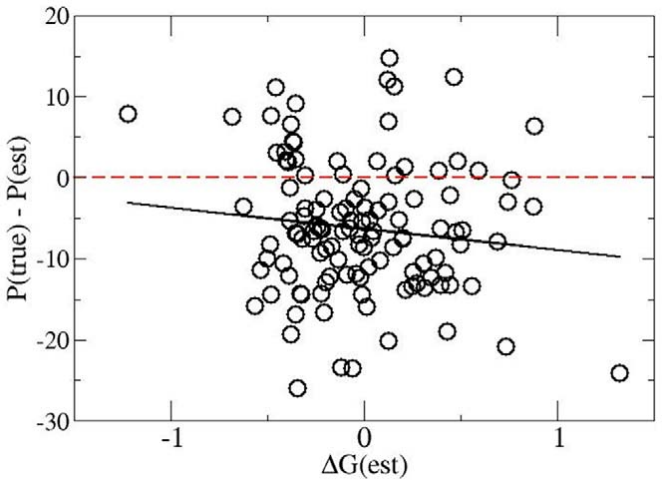
Correlation of the deviation from the expectation of points with the expected fitness in a season where a CD takes please. The solid line is the regression line. From this graph the motivation to dismiss a coach can be extracted.

The results are displayed in Fig. 6. Obviously, most (82%) of all teams have indeed performed worse than the pre-season expectation. Thus, the motivation to dismiss a coach is not only pure imagination but is indeed backed by a bad performance (which, unfortunately, does not change after the CD). Interestingly, the deviations from expectation are stronger for good teams (on average up to 9 points for the whole season) as compared to bad teams with approximately half of the number of points. This may have a simple psychological explanation. Even with a somewhat poorer performance good teams are still significantly distant from the relegation positions. Thus, for these teams the need for action results from the mere comparison with the expected outcome. For bad teams, however, already a minor negative deviation will push these teams to positions very close to relegation. This may immediately increase the pressure to act and thus to dismiss the coach as the most simple action.

We have repeated the analysis with evaluating the number of points after midseason, i.e.at the average time of the coach dismissal. The graph looks similar albeit with slightly smaller values for the number of points (because only half of the season is over). In any event, the interpretation remains exactly the same as before.

### CC: Analysis of Possible Effects

Having found no signature of the in-season CDs one may wonder whether changing the coach during the summer break, i.e. a CC, has an influence on the team performance. This question has two facets. First, independent of the quality of the coach the mere act of changing a coach may bring in a systematic shift in fitness. Of course, this shift may be positive (e.g. due to bringing in new stimulus in saturated structures) or negative (e.g. due to corrosion of well-established team structures). Second, beyond this systematic effect the different qualities of coaches might lead to the effect that some teams profit whereas other teams may suffer from this change (relative to the average). Whereas the systematic effect can be studied from the first moment of the appropriate performance distribution, the variance of this distribution contains additional information about the quality variation of different coaches, as already discussed in the context of CD.

**Figure 7:**
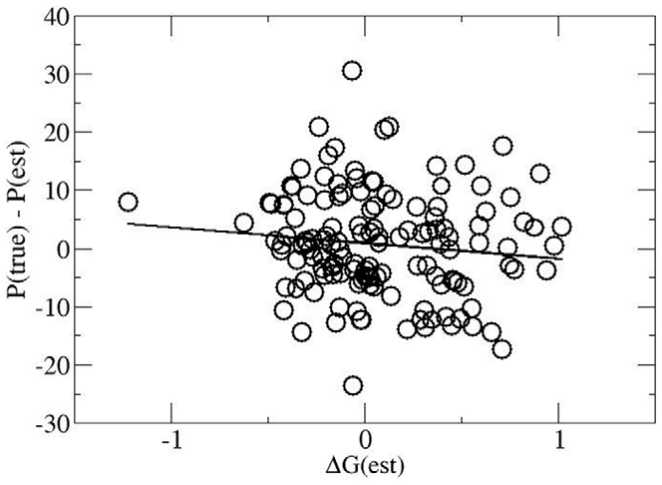
Correlation of the deviation from the expectation of points with the expected fitness in a season where the coach has been changed in the previous summer break. The solid line is the regression line. From this graph the effect of changing a coach in the summer break can be extracted.

In analogy to above we start by correlating P(true) – P(est) with ΔG(est); see Fig. 7. It turns out that the average value of P(true) – P(est) is −0.3±0.6. Thus, no significant overall improvement of deterioration is seen. Furthermore, no significant correlation with ΔG(est) is observed since the relative error of the slope of the regression line is approx. 70% of the slope itself. Thus we may conclude that a possible systematic effect of a CC is less than one point per season, i.e. totally negligible. Repeating the same analysis for ΔG(true) - ΔG(est) (as before defined as the average goal difference per match) we obtain −0.02±0.04 which again indicates that any effect, if present at all, is very small. We may conclude that changing the coach has no systematic positive or negative effect.

In the next step we study the variance of ΔG(true) - ΔG(est) of the CC teams. In what follows we restrict ourselves to the distribution of goal differences due to its superior properties as compared to the number of points. For the variance we obtain the value of 0.197±0.026. Here the statistical error is smaller than in the CD analysis because we include information from a complete season rather than just from 10 matches. To identify the statistical contribution (due to the random effects in a soccer match beyond the actual team fitness) we also determine the variance for all teams. We take the same seasons as for the CC teams and, of course, also require that the team was playing in the Bundesliga in the previous season (for the determination of ΔG(est)). Here the variance is given by 0.212±0.013. The difference of the variances thus reads −0.015±0.029. Within the statistical error no difference to the variance of the CC teams is present. Note that a significant quality variation among the coaches would have resulted in a positive value of that difference. In any event, the hypothesis that all coaches basically have the same or similar quality (or their quality is irrelevant for the team performance) and that a CC has no direct effect cannot be ruled out by studying the data of more than 40 years Bundesliga.

Taking into account the size of the statistical error one may estimate the possible relevance of the specific coach on the team performance. With an optimistic view the maximum increase of the variance is given by −0.015+2×0.029≈0.04. The value has to be compared with the fitness variance of all teams in the Bundesliga which is 0.27 (see above). This implies that with this optimistic estimation the relative contribution of the coach to the team fitness is 0.04/0.27, i.e. 15%. Most likely, however, this contribution is even smaller. This small value also reflects the fact that the group of coaches, which is considered to be hired in the Bundesliga, fulfills already high quality criteria so that the quality variation within this group is quite small.

## Discussion

This work can support the results of some previous studies [11]–[15], but now ruling out several methodological weaknesses and covering a very large data set with respect to effects of coach dismissals. The underlying team fitness does not improve due to coach dismissal. The increase immediately after the coach dismissal can be completely traced back to a simple statistical selection effect (regression towards the mean). The idea to dismiss a coach emerges from a bad performance as compared to expectation (see Fig. 6) and the actual dismissal is triggered by two particularly unfortunate matches. Furthermore, for teams below the average a smaller deviation from the pre-season expectation may be sufficient to dismiss the coach as compared to better teams where typically a larger deviation is required.

Changing the coach during the summer break results in the same nil effect. Most interestingly, even the variance of the appropriate distribution of teams changing the coach during two seasons does not show any effect. This has the immediate consequence that the impact of coaches as “fitness producers” for the teams is limited and is most likely (on average) much smaller than 15% as compared to other factors (like the team wage bill [18]), determining the quality of a soccer team. Stated differently, the quality of coaches, working in the Premier German Soccer league and hired successively by a team is either quite similar or does not have much impact on the quality of the team as already assumed before [3]. Our results do not exclude the possibility that it is favorable to work with a coach several years in a row. This aspect will be studied in future work along similar lines.
